# Massive production of fibroin nano-fibrous biomaterial by turbulent co-flow

**DOI:** 10.1038/s41598-022-26137-7

**Published:** 2022-12-19

**Authors:** Alfonso M. Gañán-Calvo, Sergio Blanco-Trejo, Miguel Ruiz-López, Gustavo V. Guinea, Luis B. Modesto-López, José Pérez-Rigueiro

**Affiliations:** 1 Departmento de Ingeniería Aeroespacial y Mecánica de Fluidos, ETSI, Universidad de Sevilla, Camino de los Descubrimientos s-n, 41092 Sevilla, Spain; 2 Laboratory of Engineering for Energy and Environmental Sustainability, Universidad de Sevilla, 41092 Sevilla, Spain; 3 Centro de Tecnología Biomédica, Universidad Politécnica de Madrid, Pozuelo de Alarcón, 28223 Madrid, Spain; 4 Departamento de Ciencia de Materiales, ETSI Caminos, Canales y Puertos, Universidad Politécnica de Madrid, 28040 Madrid, Spain; 5 Biomedical Research Networking Center in Bioengineering, Biomaterials and Nanomedicine (CIBER-BBN), 28049 Madrid, Spain

**Keywords:** Fluid dynamics, Chemical biology, Biomaterials

## Abstract

Among the different polymers (proteins, polysaccharides, etc.) that make up natural fibers, fibroin is a protein produced by silk spinning animals, which have developed an optimized system for the conversion of a highly concentrated solution of this protein into high-performance solid fibers. This protein undergoes a self-assembly process in the silk glands that result from chemical gradients and by the application of mechanical stresses during the last step of the process. In the quest for a process that could mimic natural spinning at massive scales, we have discovered that turbulence offers a novel and promising solution: a turbulent liquid jet can be formed by a chemically green and simple coagulating liquid (a diluted solution of acetic acid in etanol) co-flowing with a concentrated solution of fibroin in water by the use of a Flow Blurring nebulizer. In this system, (a) the co-flowing coagulant liquid extracts water from the original protein solution and, simultaneously, (b) the self-assembled proteins are subjected to mechanical actions, including splitting and stretching. Given the non-negligible produced content with the size and appearance of natural silk, the stochastic distribution of those effects in our process should contain the range of natural ones found in animals. The resulting easily functionalizable and tunable one-step material is 100% biocompatible, and our method a perfect candidate to large-scale, low-cost, green and sustainable processing of fibroin for fibres and textiles.

## Introduction

The spinning of silk fibers by spiders and silkworms^1^ represents a paramount example of conversion of a macromolecular solution into a solid—high peformance—material. The whole spinning process is underpinned by a series of singular features that may be identified at several levels, starting from the organization of the genome, which include protein expression, to the physiological mechanisms that finally lead to the assembly of the fiber^2^.

Silk fibers are constituted by a family of proteins called fibroins (or spidroins in the case of proteins expressed by spiders^3–5^). All fibroins share a common design in which a central region, with a large number of repetitions of a few short motifs of sequence, is flanked by specific motifs at the N- and C-termini^6^. The N- and C-termini are highly conserved along evolution and play an essential role in the initial steps of the self-assembly process undergone by the proteins in the silk gland^7,8^. Subsequently to the initial assembly of the proteins in the gland, the completion of the spinning process requires the formation of $\beta$-nanocrystals^9,10^ that result from the piling up of $\beta$-pleated sheets both in fibroins and spidroins. The $\beta$-nanocrystals are formed by motifs that appear in the central repetitive region of the fibroins, which include the -GAGAGS- motif in the silkworm *Bombyx mori* (*B. mori*) silk and the polyalanine (-An-) motif in major ampullate gland silk^3,5^.

The formation of the $\beta$-nanocrystals is controlled by mechanical stresses exerted on the liquid phase constituted by the self-assembled fibroin proteins^11,12^, which allow the adaptation of the material to the immediate requirements of the spinning organism^13^. The rheology of the fibroin solutions, in combination with the geometry of the silk gland and the measured spinning speeds, imply that the natural spinning process proceeds under conditions of laminar flow^14,15^, where stresses of approximately 1 MPa are estimated to be required for the formation of the nanocrystals^16^. The application of these stresses takes place while the protein solution is homogeneously elongated through the animal’s dragline spinneret, the pH is modulated to provide the adequate hydrogen bonds that facilitate the formation of the crystals, and water is extracted from the solution^17^. Thus, the relatively large stresses of 1 MPa (from two to three orders of magnitude smaller than the final fiber resistance, though) imply a complex but gentle process where the fundamental energy per unit volume is provided by the external pull on the outer fiber created by the animal movements. The animal’s internal organs gently respond to the external material and mechanical demands towards an evolutionary optimum in the mechanical performance of the fiber. Following this rationale, essentially all proposed spinning processes which intend to produce fibers from fibroin solutions are designed to follow a laminar flow regime^18–20^. However, the intrinsically evolutionary idea of the fiber drawing by arthropods leaves an open question: starting from a concentrated solution of fibroin as that internally found in weaving arthropods, is it possible to massively produce a fibrous material by stochastic yet gentle processes that may resemble the natural ones in any way?

In this work, we provide a rather simple affirmative answer to that question: we show that it is possible to mass-produce a tangled fibrous material consisting of silk fibers from a concentrated solution (around 30 wt%) using the hydrodynamic conditions within a turbulent regime. We show that even though the kinetic energy rate $\varepsilon = U^3/D$ is such that the turbulent energy spectrum $E\sim \varepsilon ^{2/3} k^{-5/3}$ yields spatially distributed stresses well below 1 MPa, an ample spectrum of fibrous structures from about tens of microns down to a few nanometers is produced, where $k>D^{-1}$ is the wavenumber, *U* the macroscopic speed of the solution, and *D* a characteristic macroscopic length (e.g. the diameter of the discharge orifice of the solution). The distribution of stresses^21^ characteristic of a turbulent regime is thus reflected in a distribution of equivalent diameters of the fibers that span about three orders or magnitude. Interestingly, despite we use mechanical pressures of the order of 0.2 MPa < 1 MPa to drive the solution and the co-flow, the entangled motions undergone by the solution exert a spectrum of local stresses while it is simultaneously strained and subjected to water extraction by the turbulent co-flow. We propose that part of this spectrum, applied to the smaller fiber diameter range, should have values above 1 MPa due to the inertial motions of large scales that somewhat resemble part of the spectrum of the external demands of a weaving animal. Analyses of the diameter distribution of the resulting fibers and their nature supports this hypothesis.

The simple mass-processing method presented herein produces fibrous structures that may be adequate for many applications under an appropriate post-processing (e.g. wet stretching, carding, bundling, drawing, yarning, etc.): as scaffolds in the context of Tissue Engineering, as base materials in biomedical composites, hypoallergenic fabrics or pads, as substrates in Controlled Drug Delivery, or as highly resistant raw material for fabrics and films. In reality, fibers are never used individually (with very few exceptions), but forming bundles, yarns or felts, which makes this method particularly suitable for large scale processing of fibroin. In addition, its simplicity allows the use of alternative dope formulations or its fine-tuning using a wide range of operating conditions.

## The fibrous bio-material

*Bombyx mori* silk fibroin was provided by Silk Biomed S.L. (Madrid, Spain). The formation of the fibers from the initial dope (with a fibroin mass concentration of 29% in ultrapure water + CaCl$_2$ 1M) follows a process by which the solvent is extracted by the surrounding environment by diffusion while it undergoes viscous stresses owing to the turbulent motion. To obtain the fibers, the dope is firstly forced through a Flow Blurring nebulizer (OneNeb, by Ingeniatrics Tec. S.L., Spain) at a rate $Q_d=2.2$ ml/min with a co-flow of 80% ethanol and 20% acetic acid 1M in ultrapure water at a constant rate $Q_c=50$ ml/min. The surrounding environment is either the bath (initially, with the same composition as that of the co-flowing liquid), the co-flowing liquid, or a mixture of both, depending on the position of the fiber along the ejection area from the nebulizer (see Fig. 3). Due to the relatively large size of the bath (10 liters) compared to the mass of dope expelled (1.5 g), the composition of the environment is assumed constant.

### Mass rate and costs compared to other methods

In our current setup, our method yields mass rates of the order of 10 to 20 mg/s through an orifice of 240 $\upmu$m (OneNeb), but it can be scaled up to yield, for example, about 0.1 to 1 g/s. through a single orifice of about 2 mm. These yields can be compared to existing silk fiber drawing means as follows.

In the natural (laminar) process, a silkworm spins a silk fiber with a typical diameter of 12 $\upmu$m (10–17 $\upmu$m) at speeds of the order of $10^ {-2}$ m/s^22^. This is about 1.5 $\upmu$g/s of silk fibroin, about 4 to 6 orders of magnitude below our method.

Besides, the “Straining Flow Spinning” (SFS, a laminar process, too) described in several works by Rigueiro, Madurga, Guinea, Gañán-Calvo et al.^23,24^, they had $Q_d=5\, \upmu$l/min or below, using fibroin concentrations of the order of 10 to 20% at most. This is 10 to 20 $\upmu$g/s, from 3 to 5 orders of magnitude lower than our method and using about ten times more reactant (co-flow of ethanol) relative to the dope rate.

Furthermore, electrospinning has also been used in silk fiber drawing. Assuming that the dope formulation is the same as the one used for SFS (fibroin in water), to produce a fiber with the same diameter as the natural one, avoiding electrical gas discharges, the electrical conductivity of the dope should be about 0.1 to 2 mS/m according to established scaling laws^25,26^, and the corresponding mass rate should be about 1 to 2 mg/s. This is from 10 to 1000 times smaller per spinneret than our method. In addition, the electrochemistry needed to tune the electrical forces for a laminar drawing without protein degradation and the use of high voltage electrical fields makes electrospinning much more tricky and costly than previous methods, let alone compared to present method.

### Turbulent process as a viable alternative

**Figure 1:**
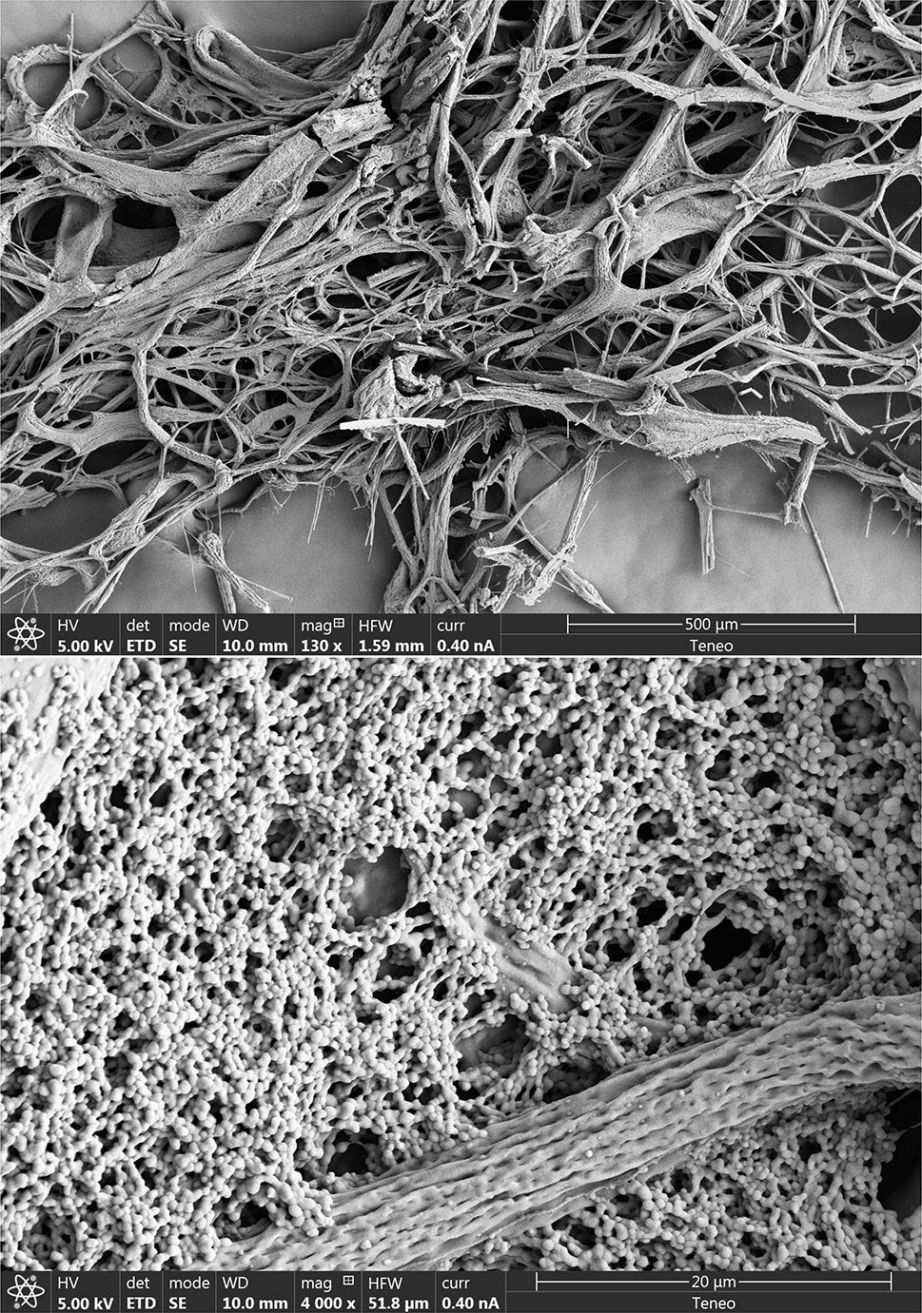
(Top panel) Result from experiment 1: the millimeter-scale appearance of the fibrous as-blown (non-stretched) material, without additional processing. Here, the absence of any additional mechanical input leaves a structure dominated by the inertia of the large scales. (Bottom panel): An illustration of the free-blowing structure of the material: the non-stretched globular structure, as described by^33^, is readily apparent together with a mildly stretched fiber that exhibits a typical micro-structure revealing its origin.

While the dope undergoes a very controlled laminar spinning in previous processes such as natural, electrospinning or SFS, in the present process it undergoes an initially strong but decaying turbulent co-flow that exerts a vigorous chaotic exchange of mass and momentum with the dope. In this process, the co-flow produces an initial mixing with the dope at the central capillary of the nebulizer according to the Flow Blurring mechanism^27–29^. The dope stream injected through that central capillary is then strained and split into a chaotic sequence of fibrous structures (Fig. 1), which are ejected with the turbulent co-flow stream discharged through the exit orifice as a turbulent liquid jet.

Along the axial, turbulent decay evolution of the co-flow jet, the dope is exposed to characteristic spectra of flow patterns (highly extensional regions, instantaneous stagnation points, branching eddies) and turbulent intensities of decaying jets^30^, nearly following the evolution of material lines^21,31,32^. The final fibers are assumed to have a uniform chemical composition (i.e. the final mass concentration of fibroin, close to 100%). This concentration obviously changes along the process due to solvent extraction by diffusion, but the mass of fibroin is conserved along the process involving stretching, folding and splitting of the fibrous structure. Two fundamental outer actions, chemical and mechanical, are applied on the dope along the turbulent process: (1) the rate of water extraction and (2) the local strain rate. Both actions compete to determine the final internal fiber structure. When water extraction is faster, the structure becomes dominated by globular geometries whose size is fundamentally determined by the protein nature and concentration^33^, see Fig. 1(bottom). In contrast, when the strain rate dominates, the water extraction merely responds to the local increase in surface area associated to the fiber stretching, and the fiber structure becomes nearly identical to that of the fibers naturally drawn by organisms. In between, both the chemical and mechanical rates homogeneously compete in the process to yield extensive characteristic repeating patterns along the fibers (see Figs. 1(bottom) and 2). These effects are readily present in the freely fiber-blowing conditions of experiment 1 (Fig. 3a) without any external or post-processing actions.

**Figure 2:**
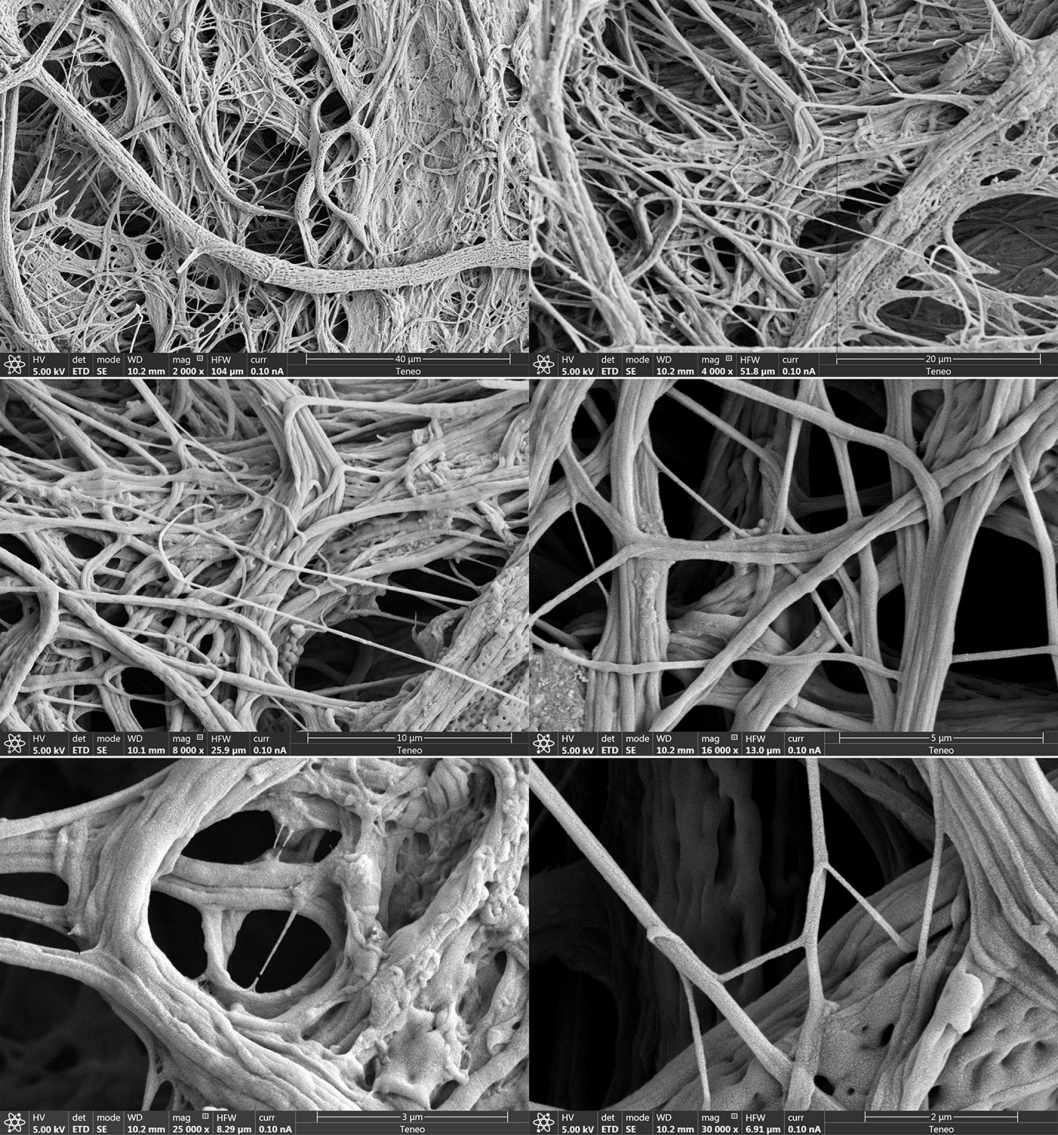
Different examples of the fibrous structure used to measure fiber diameters and lengths, for five zoom-in levels.

**Figure 3:**
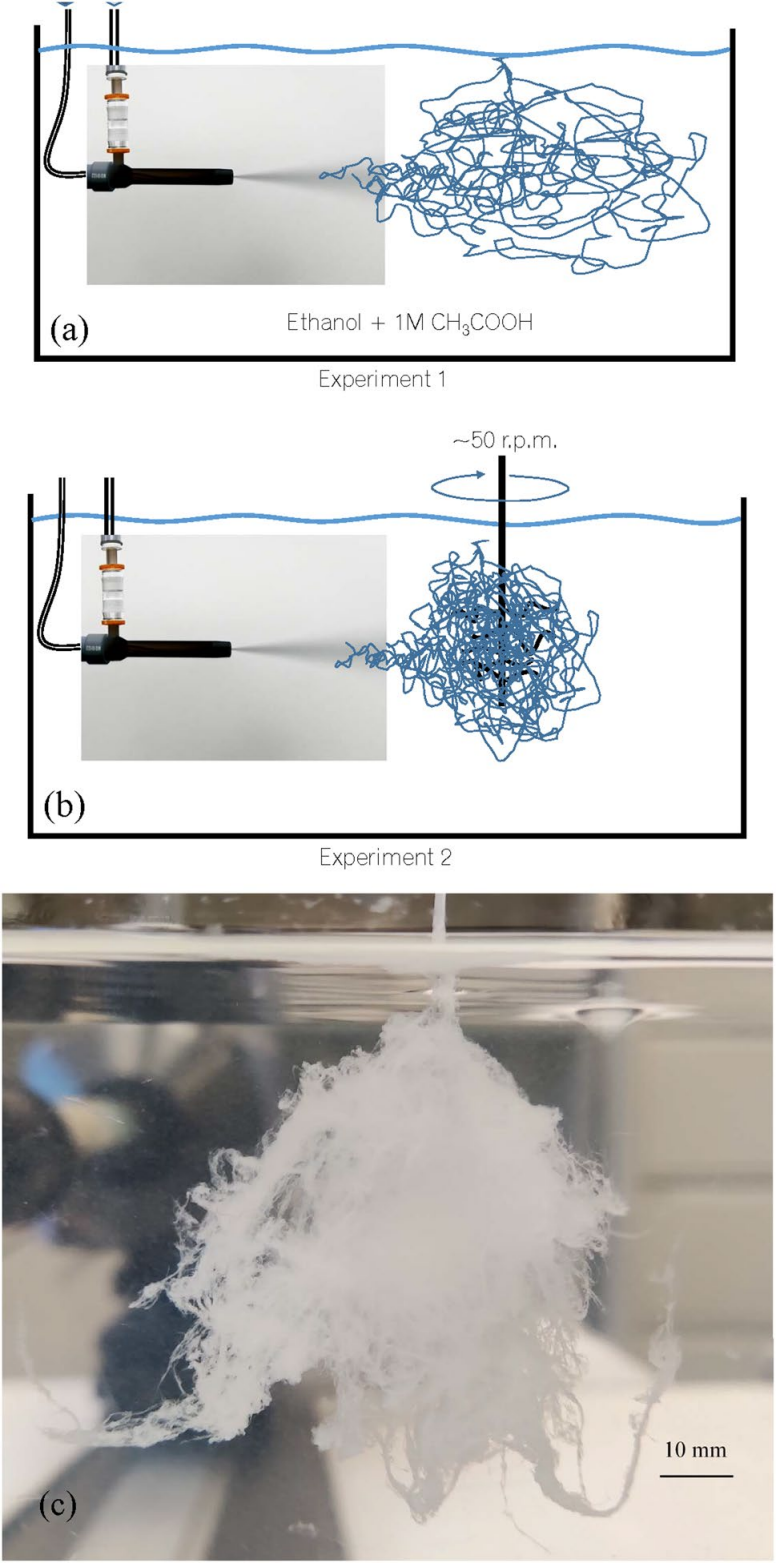
(**a**) Experiment 1, fiber free-blowing process. (**b**) Experiment 2: fiber blowing with collection. (**c**) The cotton-like fibrous material collected from a single 25-s ejection of 1.5 g of the fibroin solution, from experiment 1 (as-blown fibers).

**Figure 4:**
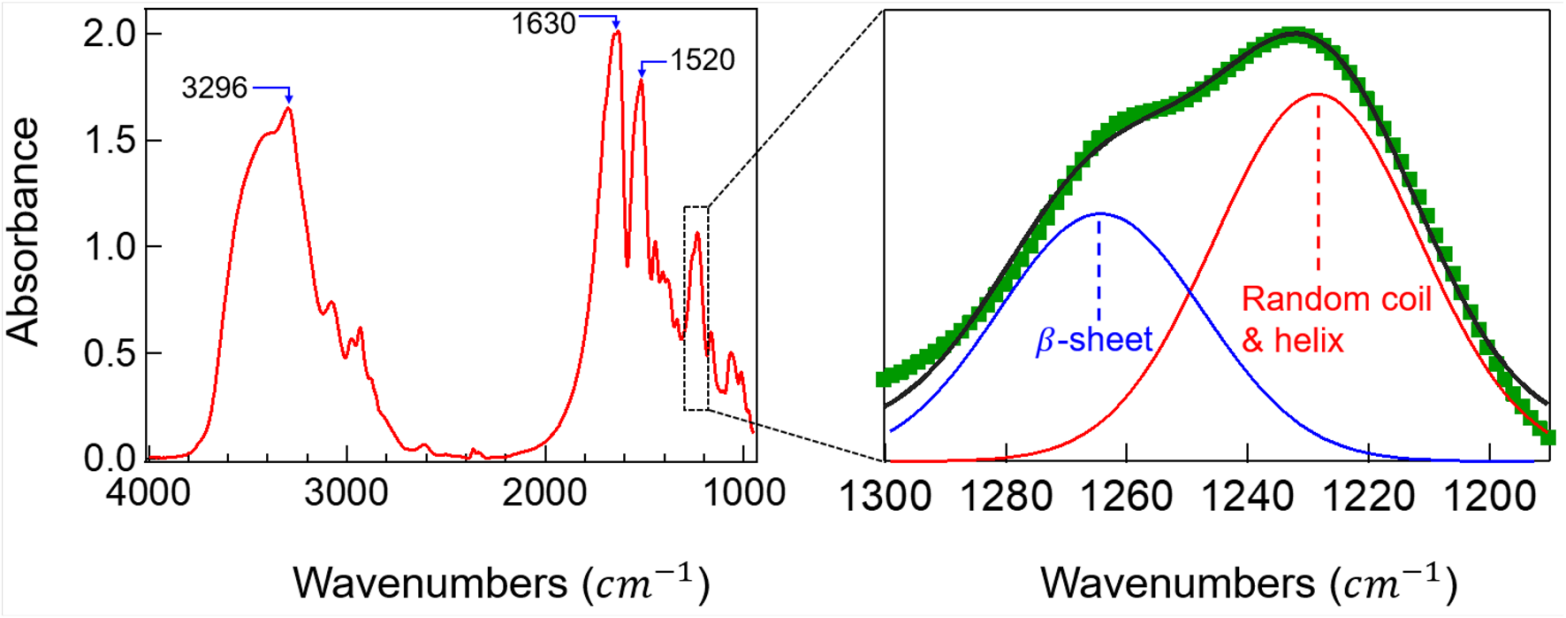
FTIR spectra of fibroin fibers produced by turbulent drawing.

The described competition only occurs if the dope concentration is sufficiently high for water extraction to be controlled by mechanical actions, at least over a significant part of the spectrum of the co-flow characteristic turbulent scales. In this case, the final structure reveals the action of the large scales over the smaller ones: the inertial motions of the former bestow the drawing action on the latter by simple drag (see the SEM micrographs in Fig. 2). This drawing action can be enhanced by collecting the cotton-like resulting material (see Fig. 3b, experiment 2) with a rotating brush, for example. The wide spectrum of scales and the inertia of the large ones guarantee that fibers with the smaller diameters undergo high and relatively homogeneous stresses along their span. Indeed, those stresses can overcome by orders of magnitude the applied macroscopic pressure that drives the combined flow of the dope and the co-flow, mostly because the ratio of the former over the latter is small (about 0.04). Furthermore, the cotton-like material produced herein shows the characteristic FTIR signatures of silk fibroin fibers^34,35^ as shown by the spectra of Fig. 4. The figure also depicts the existence of both structures, $\beta$-sheet and random coil and helix, characteristic of the naturally drawn fibers. Thus, the product is a viable fibrous material with the same basic components of natural fibers, which makes it a perfect base material for further processing to consolidate molecular structures along predefined directions (e.g. wet stretching), reduce brittleness and enhance substantially its mechanical resistance.

The resulting microscopic fibrous structure exhibits clearly identifiable fibers with a relatively constant diameter (typical deviations below 20% along the fiber length), especially those with diameters below 1 micrometer. Each individual fiber can be defined by a set of diameter measurements along its axis, and a set of points defining its axis from one end to the other which are normally determined by bifurcations. In addition, some fibers present a flatten cross section, especially those with apparent diameters beyond 10–20 $\upmu$m.

## Experimental characterization

Figure 2 gives some examples of the 17 SEM images used in this study. The average diameter is $\langle d \rangle = 413$ nm, while the average length results around $\langle l\rangle \sim 10$ micrometers, obtained as follows.

Using the simple measurement procedure described in section “Methods”, we have identified 620 fibers from our experiment No. 2 (where fibers are collected by a rotatory brush). The statistics of the fiber diameter is described next.

### Fiber diameter statistics

**Figure 5:**
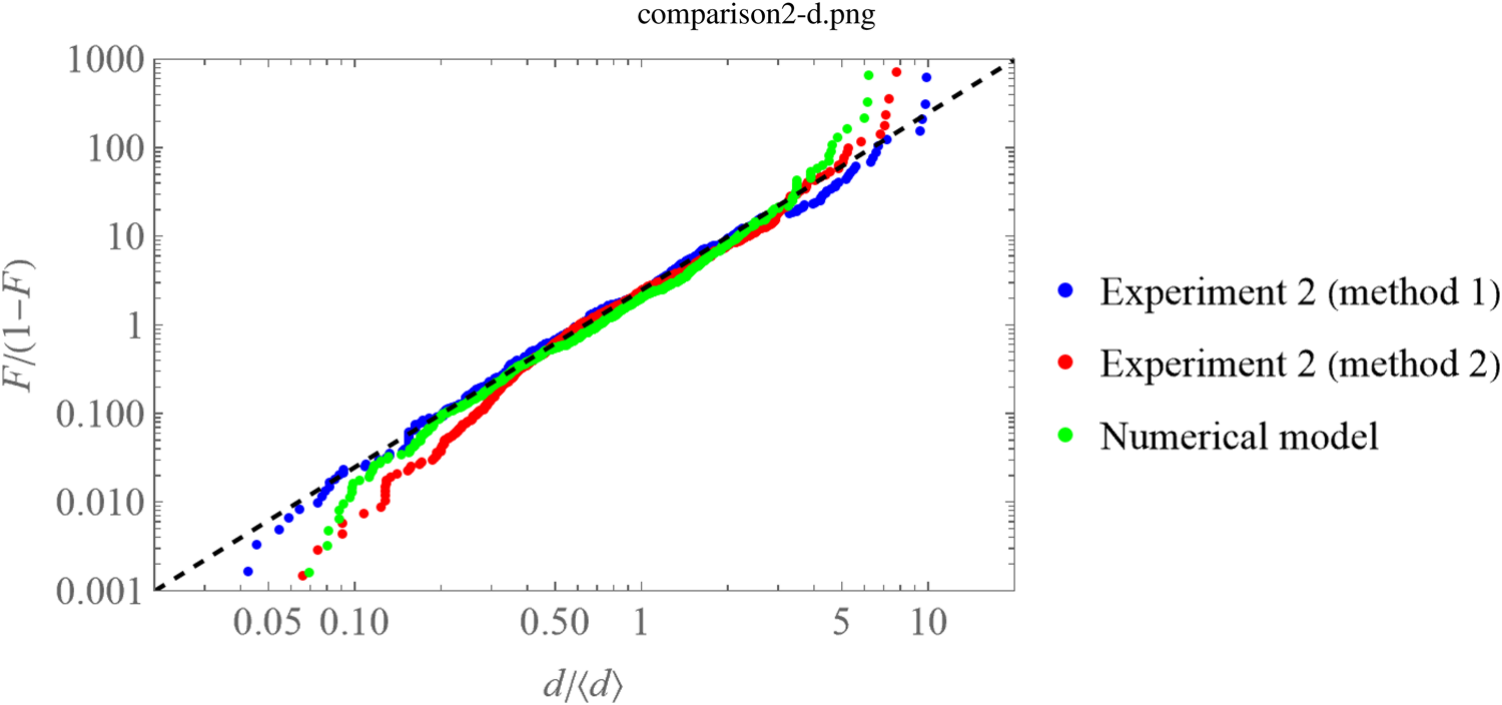
Function $F/(1-F)$, where *F* is the cumulative distribution function of the corresponding stochastic variable $x=d/\langle d\rangle$ (normalized fiber diameter). The experimentally measured values (620 data) are compared to the results of the numerical simulation according to the proposed model (see text).

Figure 5 shows the function $F/(1-F)$, where *F*(*x*) is the cumulative distribution function of the normalized fiber diameter $x=d/\langle d\rangle$, where $\langle \rangle$ means mean values.

Interestingly, the data are very well fitted by the simple power-law function $F/(1-F)=(\pi x/2)^2$, corresponding to the analytical probability distribution function $f(x)=dF/dx= \pi (\pi x/2)/\left( 1+(\pi x/2)^2\right) ^2$. This functions exhibits power-law behavior at both $x>>1$ and $x<<1$ ends, as expected from finite-time stochastic fragmentation and stretching processes^36^, in contrast with the log-normality of the length of material lines described in^32^. However, the stretching of individual fibers between successive splits may be expected to follow the log-normality predicted by those established models^32^, although the appearance of power-law tails cannot be ruled out^21^, in particular when the concentration of the dope fibrils begins to increase.

While the reliability of the fiber length becomes compromised for lengths over the limit size of the SEM frames, the fiber diameters are very well-repres ented statistically. The experimental values of the normalized fiber diameter $x=d/\langle d\rangle$ are compared to the results of a numerical model outlined below.

### Mechanical significance of the mathematical scaling of the fiber diameter spectrum

Having the cumulative distribution function of the normalized diameter $x=d/\langle d\rangle$ obeying $F/(1-F)=(\pi x/2)^2$ means that the probability distribution function (p.d.f.) *f*(*y*) of the fiber cross section $y=x^2$, i.e. $f(y)=d F/dy$, is a constant for fiber diameters below its mean value. On the other hand, the large size side of the spectrum decays faster than $y^{-2}$ (in reality, it does that in an exponential fashion for the largest sizes, see Fig. 5), indicating that the span of the large sizes is rather limited.

First, to have a limited range of large sizes imply that those larger fibers, which generate dominant stresses along their evolution over all smaller section fibers stuck or entangled to them, will produce a limited range of stresses, too. This is what the SEM images of the entangled fibrous material suggest. In addition, given that the probability distribution of cross sections is nearly homogeneous for sizes below the mean value, those stresses are globally applied on a spectrum of fibers whose cross section is homogeneously distributed along at least two orders of magnitude. Assuming that the large scales generate forces comparable to the 0.2 MPa externally imposed values, this yields stresses up to two orders of magnitude larger, i.e. in the range up to 20 MPa for the smaller fiber size range, thus explaining its straightness, uniformity and surface smoothness compatible with the natural fibers.

The detailed physical process which yields the described remarkable property can be reduced to a relatively simple branching model as follows, which provides a universal, understandable and easily extendable explanation to the general procedure preliminarily described in this work.

## Theoretical model

We assume that the initial ejection comprises a sequence of fibers of a stochastic initial diameter around values $d_0$ consistent with the conservation of mass and the relative fraction of ejected dope and co-flowing liquid flow rates, which gives

1 $$\begin{aligned} d_0=\left( Q_d/Q_c\right) ^{1/2}D. \end{aligned}$$

The initial fiber segments ejected undergo a series of stretching and splitting steps as they are passively conveyed by the turbulent co-flow. Details of the model are given in section “Methods”. There are two implicit degrees of freedom associated to the branching mechanism of each splitting event which define the length of each resulting fiber, and one restriction due to conservation of mass. In this preliminary study, we have not attempted to model the fiber length since our experimental length measurements are not as reliable as those of the diameter. Each parameter affects an aspect of the final p.d.f. as well as the final average value $\langle d\rangle$. The first fundamental observation is that the p.d.f. approaches log-normality as the number of splits *m* increases over 4, while it tends to a power-law cumulative distribution *F*(*x*) when $1<m\le 4$, such that

2 $$\begin{aligned} h = F/(1-F) = \left( \frac{\pi x}{\alpha \sin (\pi /\alpha )}\right) ^\alpha \Rightarrow F=\frac{\left( \frac{\pi x}{\alpha \sin (\pi /\alpha )}\right) ^\alpha }{1+\left( \frac{\pi x}{\alpha \sin (\pi /\alpha )}\right) ^\alpha }, \end{aligned}$$

with $$\alpha >1$$.

### Model comparison

The representation (2) is particularly useful when power-law tails are expected since it immediately reflects this behavior. From the experimental measurements, plotting the values $h(x_i)=i/(M+1-i)$ as a function of the sorted values $x_i$ of each measured *x* variable, one obtains the plot in Fig. 5, where *M* is the number of fibers measured. The experimental measurements can be fitted by a power law with $\alpha =2$. That behavior is obtained by numerical simulation following the algorithm described in Methods (“Fiber splitting and stretching model”), when $\alpha _d/\beta _d\simeq 0.7$ and $\alpha _d \in (0.03,0.1)$, with deviations occurring for the last 1% at both $x\ll 1$ and $x \gg 1$ extremes. In Fig. 5, the number of splits is set to $m=4$, and $\alpha _d=0.05$, $\beta _d=0.07$. The value of $\langle \zeta \rangle = 4.5$ of the stretching determines the average $\langle d\rangle$ experimentally measured, with a very small sensitivity to the choice of Var$(\zeta ) \in (0.1,3.0)$ except at the 1% extreme values. In Fig. 5 we have chosen a variance Var$(\zeta )=1.5$.

**Figure 6:**
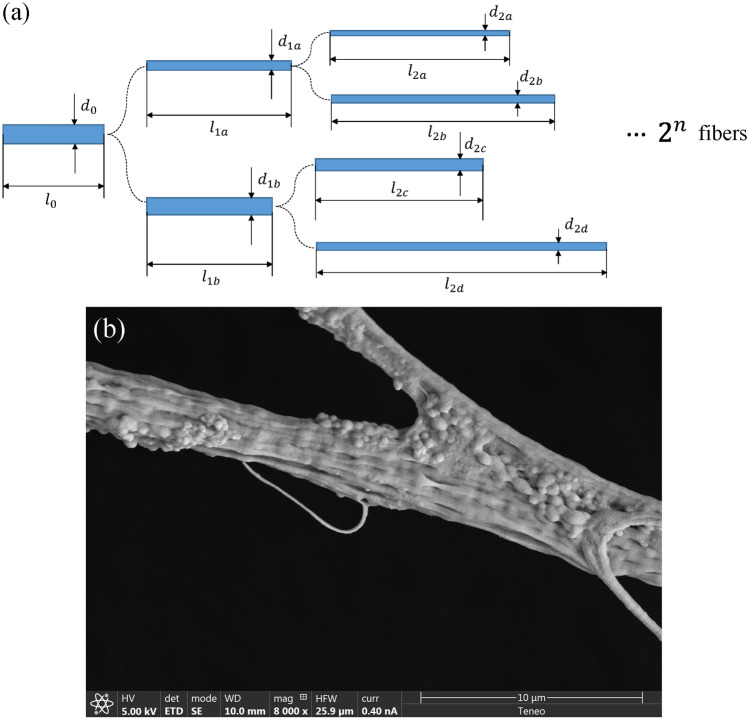
(**a**) Branching algorithm here proposed for each initial fiber: it splits and stretches following fibroin mass conservation. Both fiber split ratio and elongation rate are randomly generated following pdfs discussed in the text. (**b**) An illustration of the fiber splitting principle used in the model here proposed: a frozen split.

The remarkable agreement found with experimental results gives confidence on the proposed model. It points to the fundamental fact that the stretching processes take place in times much larger than the splitting, consistently with the fact that the cross section area of the fiber is preserved while it splits. In Fig. 6b a halted splitting event is shown. The process undergone by the fiber location was slow enough to allow complete solvent extraction while the stress was as mild as to allow the appearance of globular structures. The fiber section seems quite flatten in the plane of the splitting and shows the cross section area preservation principle used in the model.

In subsequent works, we will undertake the mathematical modeling of the length splitting and stretching mechanism, which are beyond the initial reporting purposes of present work.

## Discusion

In this work we show how silk fibroin fibers can be produced massively through a one-step process based on the creation of a turbulent flow, instead of the laminar flow found in the silk glands of the spinning organisms. To achieve that, we subject a fibroin solution in water with a concentration close to that found in nature to a highly efficient turbulent motion through a Flow Blurring nebulizer. Both the protein solution and of the co-flow (and coagulating) fluids require exclusively extremely mild and environmentally friendly chemistries. The turbulent motions generate a cascade of bifurcations together with additional fiber stretching, while water is extracted from the protein solution to the coagulating environment. The fibers show a distribution of diameters that spans two orders of magnitude and whose cumulative distribution function follows a remarkable simple mathematical structure. In addition, the cumulative distribution function is obtained theoretically by assuming that each fiber results from 3-4 splitting process during its formation, and that the resulting fiber diameter after each splitting step follows a Beta function distribution between 0 and $2^{-1/2}$ times the fiber diameter before splitting. In this regard, the proposed mechanism implies that fibers below the mean size should undergo nearly uniform stresses from relatively narrow-ranged large scales, which would play the role of the large scale drawing motions in the silk gland. In addition to proving that silk fibers may be produced under hydrodynamical conditions extremely different to those found in the natural spinning system, this process offers an enormous advantage for the massive production of biocompatible fibrous structures based on silk. Thus, this process allows producing a 100% biocompatible material that may be used for example as scaffold in Tissue Engineering following an absolutely green and sustainable process.

## Methods

### Measurement method

In this preliminary study, we have chosen a simplified yet sufficiently descriptive and quantitative approach for fiber characterization: the diameter of the fibers in the images is determined following two similar approaches as follows:

#### Method 1

- Each fiber is defined by an average diameter *d* of four points along its axis: two central points and two ends.
- The position of the central points is chosen at the two locations where (1) the curvature is maximum, and (2) both points are separated at least 5 diameters, except in cases where the fiber slenderness *l*/*d* is below 20. When the fiber is straight, each central point is set equidistant from the others.
- In relatively frequent cases, the fiber ends beyond the frame limits. In cases where we have taken two or more alternative SEM micrographs with different zooming, and the fiber can be identified in all them, the axial points are determined on the outmost zoom picture. However, this is not possible in most cases, and consequently the fiber length statistics is less reliable at the large-side of the size spectrum.

#### Method 2

- The algorithm of Canny^37^ is employed for a systematic edge detection on the SEM fiber images.
- Given that the fiber surface exhibits a repeated patterning, a manual selection of fiber location on the image is made.
- Three diameter measurements are made automatically using three perpendicular segments to the fiber axis. Those segments are located at the center of the fiber and the two extremes (e.g. branching points, or the edge of the image). The length of the segments is determined between the two detected edges of each located fiber.
- Three measurements are identified as belonging to a fiber if the variation coefficient between the three measurements is below 10%.

The results of these methods are plotted in Fig. 5.

### Fiber splitting and stretching model

The model assumes a splitting-stretching process as follows:

- The splitting is assumed to follow a Beta function between 0 and $2^{-1/2}$. To do so, a random number $\zeta$ homogeneously distributed between 0 and 1 is transformed into another random number $\xi =I_\zeta (\alpha _d,\beta _d)/2^{1/2}$ between 0 and $2^{-1/2}$, where $I_\zeta (\alpha _d,\beta _d)$ is the regularized incomplete Beta function. The free parameters $\alpha _d$ and $\beta _d$ determine the degree of non-homogeneity of the $\xi$-distribution. Thus, assuming the average conservation of the cross section area of the fiber before splitting, the two resulting fibers from the $i$th split will have diameters as:
  3 $$\begin{aligned} d_{i,a}=d_{i-1,a}\xi ,\quad d_{i,b}=\left( d_{i-1,a}^2-d_{i,a}^2\right) ^{1/2} \end{aligned}$$
- The stretching between two successive splits is assumed to follow a log-normal distribution^32^.
- An aggregate bunch of final fibers is generated from a number *N* of runs. Each run comprises $m-$subsequent splits as sketched in Fig. 6.

## Data Availability

The datasets used and/or analysed during the current study are available from the corresponding author on reasonable request.

## References

[1] Heim M, Keerl D, Scheibel T (2009). Spider silk: From soluble protein to extraordinary fiber. Angew. Chem. Int. Ed. Engl..

[2] Andersson M, Johansson J, Rising A (2016). Silk spinning in silkworms and spiders. Int. J. Mol. Sci..

[3] Xia Q (2004). A draft sequence for the genome of the domesticated silkworm (*Bombyx mori*). Science.

[4] Xu M, Lewis R (1990). Structure of a protein superfiber: Spider dragline silk. Proc. Natl. Acad. Sci. USA.

[5] Gatesy J, Hayashi C, Motriuk D, Woods J, Lewis R (2001). Extreme diversity, conservation, and convergence of spider silk fibroin sequences. Science.

[6] Babb P (2017). The nephila clavipes genome highlights the diversity of spider silk genes and their complex expression. Nat. Genet..

[7] Hagn F (2010). A conserved spider silk domain acts as a molecular switch that controls fibre assembly. Nature.

[8] Askarieh G (2010). Self-assembly of spider silk proteins is controlled by a pH-sensitive relay. Nature.

[9] Riekel C (1999). Aspects of X-ray diffraction on single spider fibers. Int. J. Biol. Macromol..

[10] Cenis J (2015). Mechanical behaviour and formation process of silkworm silk gut. Soft Matter.

[11] Iizuka E (1985). Silk thread: Mechanism of spinning and its mechanical properties. Appl. Polym. Sympos..

[12] Ortlepp C, Gosline J (2004). Consequences of forced silking. Biomacromolecules.

[13] Madsen B, Shao Z, Vollrath F (1999). Variability in the mechanical properties of spider silks on three levels: Interspecific, intraspecific and intraindividual. Int. J. Biol. Macromol..

[14] Kojić N, Bico J, Glasen C, McKinley G (2006). Ex vivo rheology of spider silk. J. Exp. Biol..

[15] Holland C, Terry A, Porter D, Vollrath F (2006). Comparing the rheology of native spider and silkworm spinning dope. Nat. Mater..

[16] Jiang P (2014). Spider silk gut: Development and characterization of a novel strong spider silk fiber. Sci. Rep..

[17] Vollrath F, Knight DP (2001). Liquid crystalline spinning of spider silk. Nature.

[18] Liivak O, Blye A, Shah N, Jelinski L (1998). A microfabricated wet-spinning apparatus to spin fibers of silk proteins. Structure–property correlations. Macromolecules.

[19] Um I (2004). Wet spinning of silk polymer: I. Effect of coagulation conditions on the morphological feature of filament. Int. J. Biol. Macromol..

[20] Zhang F (2015). Regeneration of high-quality silk fibroin fiber by wet spinning from CaCl2-formic acid solvent. Acta Biomater..

[21] Davoudi J, Schumacher J (2006). Stretching of polymers around the Kolmogorov scale in a turbulent shear flow. Phys. Fluids.

[22] Miura M, Takahashi E, Sugiyama H, Morikawa H (1993). A method for measuring the spinning speed of silkworms. J. Seric. Sci. Jpn..

[23] Madurga R (2017). Production of high performance bioinspired silk fibers by straining flow spinning. Biomacromolecules.

[24] Pérez-Rigueiro J (2018). Straining flow spinning of artificial silk fibers: A review. Biomimetics.

[25] Gañán-Calvo AM (2004). On the general scaling theory for electrospraying. J. Fluid Mech..

[26] Gañán-Calvo A, López-Herrera J, Herrada M, Ramos A, Montanero J (2018). Review on the physics of electrospray: From electrokinetics to the operating conditions of single and coaxial Taylor cone-jets, and ac electrospray. J. Aerosol Sci..

[27] Gañán-Calvo AM (2005). Enhanced liquid atomization: From flow-focusing to flow-blurring. Appl. Phys. Lett..

[28] Modesto-López L, Pérez-Arjona A, Gañán-Calvo AM (2019). Flow blurring-enabled production of polymer filaments from poly(ethylene oxide) solutions. ACS Omega.

[29] Ramos-Escobar A, Uceda-Gallegos R, Modesto-López L, Gañánn-Calvo A (2020). Dynamics of formation of poly(vinyl alcohol) filaments with an energetically efficient micro-mixing mechanism. Phys. Fluids.

[30] Pope SB (2000). Turbulent Flows.

[31] Batchelor GK (1952). The effect of homogeneous turbulence on material lines and surfaces. Proc. R. Soc. Lond. A Math. Phys. Sci..

[32] Girimaji SS, Pope SB (1990). Material-element deformation in isotropic turbulence. J. Fluid Mech..

[33] Jin H-J, Kaplan DL (2003). Mechanism of silk processing in insects and spiders. Nature.

[34] Fang G (2016). Insights into silk formation process: Correlation of mechanical properties and structural evolution during artificial spinning of Silk fibers. ACS Biomater. Sci. Eng..

[35] Hu X, Kaplan D, Cebe P (2006). Determining beta-sheet crystallinity in fibrous proteins by thermal analysis and infrared spectroscopy. Macromolecules.

[36] Deane GB, Stokes MD (2002). Scale dependence of bubble creation mechanisms in breaking waves. Nature.

[37] Canny J (1986). A computational approach to edge detection. IEEE Trans. Pattern Anal. Mach. Intell. (PAMI).

